# Case Report: Two cases of atypical neurofibroma neoplasm of uncertain biological potential in the nasal cavity and a literature review

**DOI:** 10.3389/fonc.2025.1539671

**Published:** 2025-04-16

**Authors:** Ziyi Zhang, Wenhui Pang, Min Chen, Yan Jiang, Yuhui Zhao, Shengyao Fu, Junfeng Wen

**Affiliations:** ^1^ Department of Otolaryngology Head and Neck Surgery, The Affiliated Hospital of Qingdao University, Qingdao, China; ^2^ Department of Medicine, Qingdao University, Qingdao, China; ^3^ Department of Operating Room, The Affiliated Hospital of Qingdao University, Qingdao, China

**Keywords:** atypical neurofibroma of uncertain biological potential, nasal cavity, malignant peripheral nerve sheath tumor, neurofibromatosis type 1, neurofibroma

## Abstract

**Objective:**

This study aims to investigate the clinical diagnosis, treatment processes, and pathological characteristics of two cases of atypical neurofibroma neoplasm of uncertain biological potential (ANNUBP) located in the nasal cavity.

**Materials and Methods:**

We retrospectively analyzed the medical history, imaging studies, pathological results, and follow-up data of two patients diagnosed with nasal ANNUBP who were admitted to Qingdao University Hospital between June and October 2023. A review of relevant literature was conducted to summarize their clinical characteristics, diagnosis, treatment, and prognosis.

**Results:**

Both patients underwent surgical treatment, with one receiving postoperative radiotherapy. Follow-up evaluations indicated that both patients experienced satisfactory recovery, with no signs of local recurrence or distant metastasis.

**Conclusion:**

ANNUBP is a recently recognized and rare subtype of neurogenic tumor. Surgical intervention is regarded as an effective treatment method. This study adhered to established treatment principles for nasal tumors and achieved satisfactory outcomes through surgical intervention and diligent postoperative follow-up.

## Introduction

Atypical neurofibroma neoplasm of uncertain biological potential (ANNUBP) is a subtype of neurofibroma officially classified by the World Health Organization (WHO) in recent years. This term was first proposed by experts at the National Institutes of Health in the United States in 2016 to delineate neurofibromas that may undergo malignant transformation into malignant peripheral nerve sheath tumors (MPNSTs) (1). Reports on ANNUBP are limited, with most cases occurring in patients with neurofibromatosis type 1 (NF1), and only a few sporadic cases documented (1). Cases of ANNUBP arising in the nasal cavity are particularly rare, often characterized by unique clinical manifestations. Here, we present two cases along with a comprehensive literature review.

## Clinical data

### Case 1

A 50-year-old female patient presented with a chief complaint of “right nasal obstruction for six months.” She reported persistent right nasal obstruction of unclear etiology, accompanied by sensory loss in the right nostril, intermittent headaches, and clear nasal discharge but denied experiencing nasal pain, sneezing, or epistaxis. Previous treatments with oral loratadine and intranasal budesonide had proven ineffective, and symptoms of nasal obstruction persisted. The patient had no significant health issues and reported no family history of similar conditions or neurofibromatosis.

**Figure 1:**
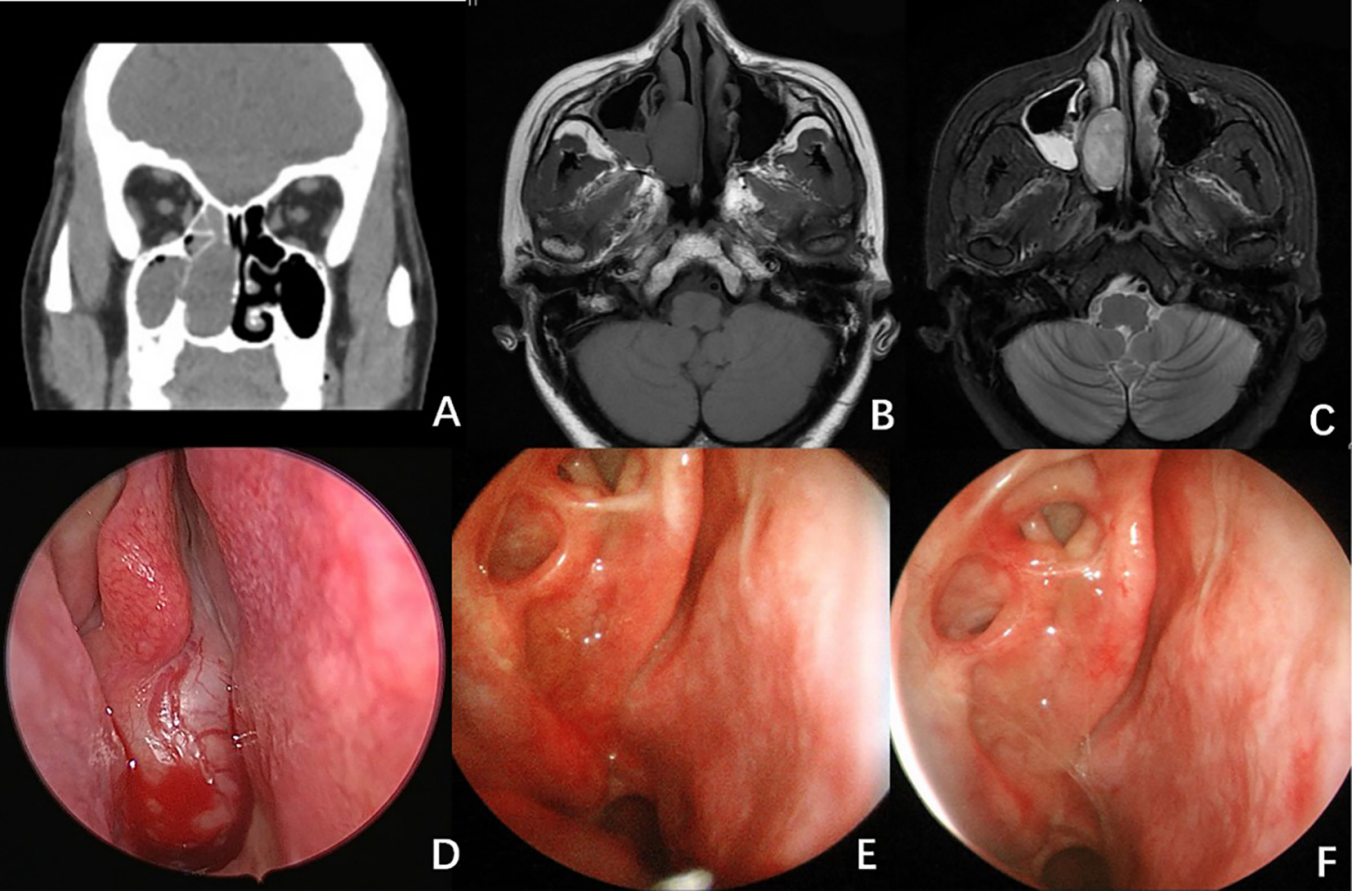
Radiological And Endoscopy Images of Case 1. **(A)** CT scan of the paranasal sinuses showed a lesion in the right nasal passage, measuring approximately 32×18 mm, with well-defined borders. **(B, C)** MRI scan showed an isointense T1 **(B)** and hyperintense T2 **(C)** signal mass in the right nasal cavity. **(D)** Endoscopy image revealed a neoplasm at the posterior end of the right nasal cavity, with purulent secretions on its surface. **(E)** Endoscopic image 5 months after surgery. **(F)** Endoscopic image 9 months after surgery. No signs of recurrence were observed.

A CT scan of the paranasal sinuses revealed a soft tissue density lesion in the right nasal cavity, measuring approximately 32 × 18 mm, with well-defined margins (see **Figures 1A, B**). An MRI scan showed an isointense T1 and hyperintense T2 signal mass in the right nasal cavity, measuring 32 × 15 × 33 mm, also with clear margins (see **Figure 1C**). Endoscopic examination revealed a neoplasm at the posterior end of the right nasal cavity, with purulent secretions on its surface.

Following comprehensive preoperative evaluations, the patient underwent surgery under general anesthesia. The tumor was excised using a low-temperature plasma system via an endoscopic approach through the middle turbinate, with electrocautery hemostasis. The tumor appeared to originate from the middle turbinate, exhibiting a smooth mucosal surface, rich vascularization, a brittle consistency, and a fleshy appearance, nearly completely obstructing the right nasal cavity while compressing local tissues (see **Figure 1D**). Intraoperative frozen section pathology indicated a spindle cell tumor, characterized by mild cellularity and few mitotic figures.

**Figure 2:**
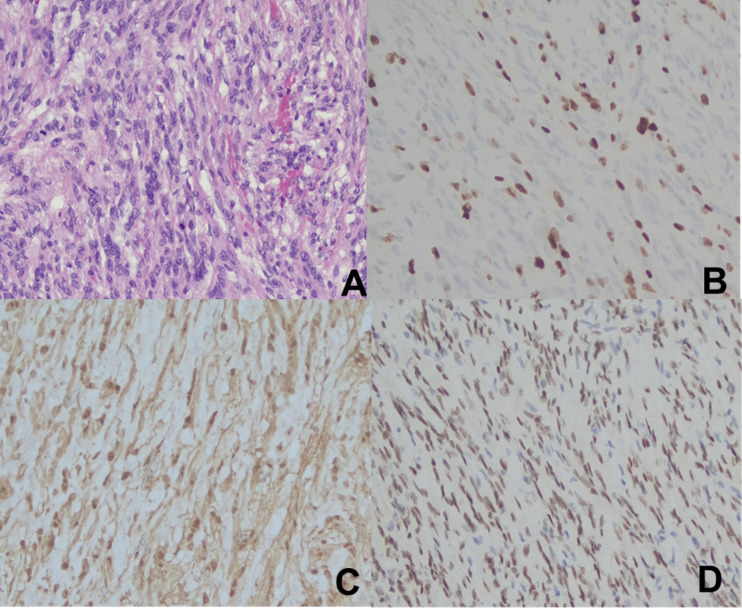
Pathological Data from Case 1. **(A)**. H-E staining (×200); **(B)**. Ki-67 (+ [hotspot region 30-40%]) (×200); **(C)**. S-100 (+) (×200); **(D)**. SOX-10 (+) (×200).

Postoperative pathology confirmed the diagnosis of ANNUBP, with certain areas showing increased cellular density and mitotic figures (4-6 per 10 high-power fields [HPF]), suggestive of focal malignant transformation to a low-grade malignant peripheral nerve sheath tumor. Immunohistochemical analysis yielded the following results: SOX-10 (+), S-100 (+), SMA (-), CD34 (-), Ki-67 (+ [hotspot region 30-40%]), STAT6 (-), pan-TRK (-), β-Catenin (cytoplasmic +), H3K27Me3 (+), HMB45 (-) (see **Figure 2**).

Radiotherapy (dose: 56Gy/28F) was administered one month after the operation. The patient was then followed up for a period of sixteen months, during which no recurrence was observed (**Figures 1E, F**).

### Case 2

A 41-year-old female patient presented with a complaint of “intermittent left nasal bleeding for four months.” She reported intermittent nasal bleeding without an apparent cause over the past four months, with small amounts of blood that ceased spontaneously. She also experienced nasal obstruction, rhinorrhea, sensory loss, and sneezing, all occurring intermittently. Previous treatment with recombinant bovine basic fibroblast growth factor was ineffective in stopping the nasal bleeding. The patient was otherwise healthy and denied any family history of similar conditions or neurofibromatosis.

**Figure 3:**
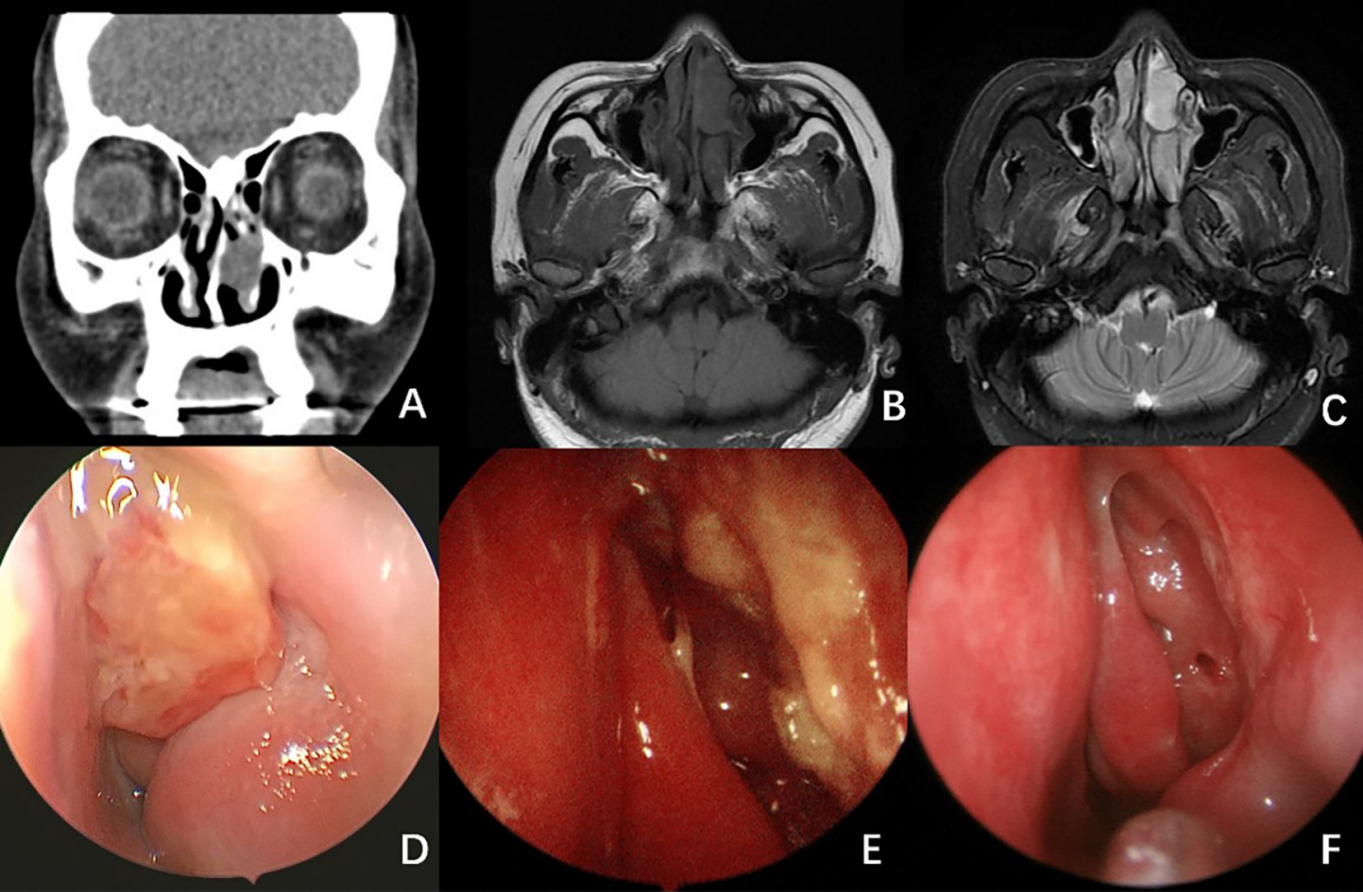
Radiological And Endoscopy Images of Case 2. **(A)** CT scan of the paranasal sinuses showed a soft tissue density mass in the left nasal cavity. **(B, C)** MRI scan showed an isointense T1 **(B)** and hyperintense T2 **(C)** nodular mass in the left nasal cavity, with relatively clear boundaries and measuring approximately 14 × 23 × 20 mm, exhibiting mostly uniform signals. **(D)** Endoscopy image revealed a neoplasm in the left nasal passage, characterized by surface vascular dilation and purulent secretions. **(E)** Endoscopic image 2 months after surgery. **(F)** Endoscopic image 5 months after surgery. No signs of recurrence were observed.

A CT scan of the paranasal sinuses indicated a soft tissue density mass in the left nasal cavity (see **Figures 3A, B**). An MRI demonstrated an isointense T1 and hyperintense T2 nodular mass in the left nasal cavity, with relatively clear boundaries and measuring approximately 14 × 23 × 20 mm, exhibiting mostly uniform signals (see **Figure 3C**). Endoscopy revealed a neoplasm in the left nasal passage, characterized by surface vascular dilation and purulent secretions.

**Figure 4:**
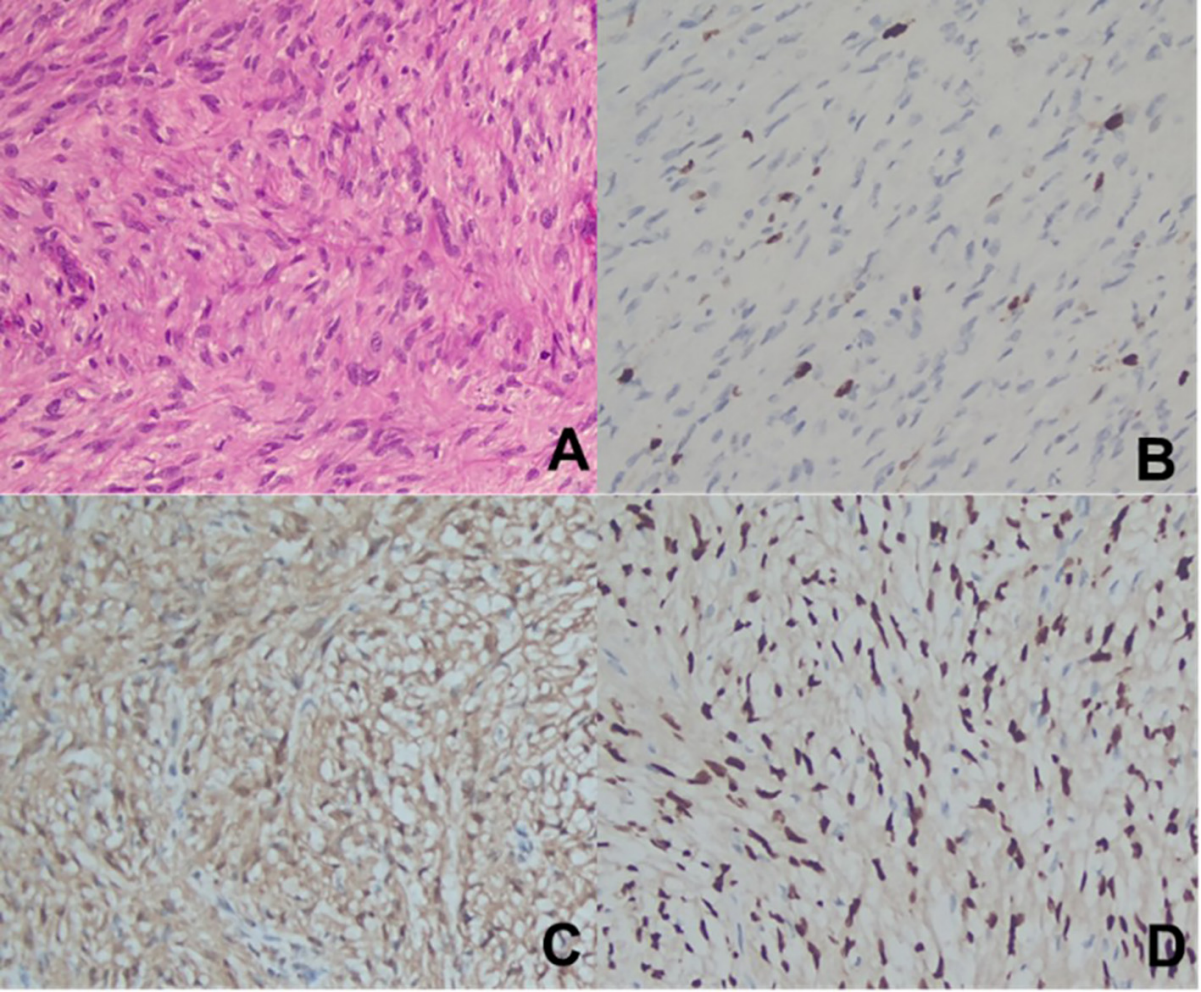
Pathological Data from Case 2. **(A)**. H-E staining (×200); **(B)**. Ki-67 (partial +) (×200); **(C)**. S-100 (+) (×200); **(D)**. SOX-10 (+) (×200).

During surgery under general anesthesia, the tumor was completely excised using an endoscopic low-temperature plasma system from the lateral wall of the nasal cavity (see **Figure 4C**). The nasal lacrimal duct opening was preserved, while part of the inferior turbinate was removed. Intraoperatively, the tumor was light yellow, featuring a granular surface and a slightly brittle consistency, occupying the anterior portion of the nasal cavity and originating from the lateral wall, involving the upper margin of the anterior portion of the left inferior turbinate. Frozen section pathology indicated spindle cell lesions with rare mitotic figures, along with interstitial edema, making it challenging to definitively exclude a diagnosis of neurofibroma.

Postoperative pathology revealed a neurogenic tumor with abundant cells exhibiting mild atypia and rare mitotic figures (< 3 per 10 HPF), which, in conjunction with morphological and immunohistochemical findings, suggested a diagnosis of ANNUBP. Immunohistochemical staining results included: S-100 (+), SOX-10 (+), GFAP (-), Ki-67 (+ [approximately 5%]), SMA (-), STAT6 (-) (see **Figure 4**).

Fifteen months post-operation, no recurrence was observed (see **Figures 3E, F**).

## Discussion

Currently, plexiform neurofibroma (pNF), atypical neurofibroma, and malignant peripheral nerve sheath tumors (MPNSTs) are recognized as part of a morphological continuum, lacking distinct boundaries that separate benign from malignant forms. The diagnostic criteria for atypical neurofibroma (ANNUBP) require the presence of a neurofibroma base alongside at least two of the following features: cellular atypia; increased cellular density; loss of neurofibroma structural integrity (e.g., fascicular growth pattern and/or absence of the CD34+ fibroblast network); and the presence of mitotic figures (≥1/50 HPF and <3/10 HPF or <1.5/mm²) (1, 2). While immunohistochemical analysis has limited utility in diagnosing ANNUBP, a complete absence of S-100 protein/SOX-10 expression and the loss of the CD34+ fibroblast network, typically found in all neurofibroma variants, may serve as useful diagnostic indicators. Although immunohistochemistry does not significantly aid in distinguishing ANNUBP from neurofibromas, varying degrees of loss in S-100/SOX-10 expression and the CD34+ fibroblast network may correlate with tumor malignancy. ANNUBP is thought to represent a precursor lesion, reflecting a transitional phase in the progression from pNF to MPNST (3).The term ANNUBP was initially introduced to refine management strategies for patients with neurofibromatosis type 1 (NF1). However, literature on sporadic cases of ANNUBP is limited, with most lesions typically found retroperitoneally, and only a few reported in the neck and upper limbs (4). Neither of the clinical presentations in the current study supports a diagnosis of NF1 (5).

MPNSTs account for approximately 5% of all malignant soft tissue tumors and may originate from peripheral nerves or develop secondarily to neurofibromas. Nearly half of MPNSTs manifest in individuals with NF1, while others are classified as sporadic or arise following radiation exposure. Many sporadic MPNSTs occur within deep soft tissues, lacking clear anatomical connections to surrounding nerves or pre-existing neurofibromas. These tumors are more frequently observed in patients aged between 30 and 50 years, whereas NF1-associated MPNSTs can occur in younger demographics, including children (6–8). The most common sites for MPNSTs include the limbs, trunk, and head and neck, with the sciatic nerve being the most frequently affected peripheral nerve, followed by the brachial plexus, sacral plexus, and paravertebral nerves. Clinically, MPNSTs typically present as palpable masses, often accompanied or unaccompanied by pain; studies suggest that NF1-associated MPNSTs have a higher propensity to cause pain (9). In patients with NF1, the rapid enlargement of existing neurofibromas warrants suspicion of malignancy. MPNSTs are categorized into subtypes based on histological traits, including (1): classic MPNSTs; (2) epithelioid cell MPNSTs; (3) perineural MPNSTs (also referred to as malignant perineurioma); and (4) malignant schwannoma melanotic tumors (10). Classic MPNSTs are the most prevalent subtype and can be further classified morphologically into low-grade MPNSTs (characterized by 3-9 mitotic figures per 10 HPF without necrosis) and high-grade MPNSTs (exhibiting >9 mitotic figures per 10 HPF or necrosis). Immunohistochemical support for MPNST diagnosis includes a Ki-67 index of >10%. Loss of CDKN2A may occur prior to the transition from neurofibroma to MPNST, while H3K27me3, commonly present in neurofibromas, including ANNUBP, is frequently lost in MPNSTs, which may assist in their differentiation. Currently, reliable biomarkers for early detection of malignant transformation in neurofibromas remain elusive (11). Some studies indicate that MPNSTs in non-NF1 patients show a greater tendency for H3K27me3 loss. High-frequency alterations in cell cycle regulators, such as P53, P16, and P27, are often found in high-grade MPNSTs but are typically absent in low-grade MPNSTs and pNFs, making these markers unreliable for early detection of MPNST (12).

The majority of MPNSTs reportedly arise “*de novo*,” presenting as highly cellular areas with diffuse cytological atypia that emerge abruptly from regions of typical neurofibromas. In contrast, the transition from typical neurofibroma to ANNUBP, and subsequently to MPNST, is relatively uncommon. Low-grade MPNSTs (approximately 10% of all MPNSTs) typically arise from NF1-related neurofibromas and can be distinguished from ANNUBP primarily based on mitotic activity, necessitating caution in diagnosis.

In terms of differential diagnosis, it is critical to distinguish ANNUBP from other neurofibroma variants, such as cellular neurofibromas, which are characterized by increased cellularity and a lack of fascicular or storiform patterns, typically presenting with rare mitotic figures. Other soft tissue tumors to consider in differential diagnoses include:

□ **Fibrosarcoma:** Microscopically, this tumor displays spindle-shaped fibroblasts intermixed with collagen and reticular fibers, which may exhibit fascicular and herringbone arrangements. It is immunohistochemically positive for vimentin and P53 while negative for SMA, CK10, EMA, CD34, and neural tissue markers (13). Low-grade fibromyxoid sarcoma may exhibit similar histology to ANNUBP but expresses MUC4, aiding differentiation (14, 15).
□ **Synovial Sarcoma:** Most cases express CD99, BCL-2, and TLE1 proteins, with molecular genetic analysis serving as a critical tool for distinguishing it from other soft tissue tumors.
□ **Melanoma:** Common markers include S-100 protein, SOX-10, Melan A, HMB45, tyrosinase, and MITF. A subset of desmoplastic melanoma exhibits neurotropic properties, complicating the differential diagnosis from neurogenic tumors (16, 17).

Both cases of ANNUBP presented in this study were located in the nasal cavity. In addition to differentiating these tumors from other soft tissue lesions, ANNUBP must also be distinguished from common nasal cavity lesions, such as:

□ **Inverted Papilloma:** A relatively common nasal tumor characterized by highly proliferative epithelial clusters demonstrating reverse growth towards subepithelial and stromal layers, associated with local invasiveness and risk of malignant transformation. Imaging may reveal a convoluted cerebriform pattern, aiding in early diagnosis.
□ **Nasal and Paranasal Hemangiopericytoma:** Typically localized to the nasal cavity and paranasal sinuses, displaying perivascular myoid differentiation, slight nuclear atypia, rare mitotic activity, and diffuse strong positivity for smooth muscle actin (SMA) exceeding that of MSA. These tumors are negative for desmin, CK, S-100, SOX-10, CD31, and ERG.

On the one hand, the diagnosis of ANNUBP relies on histopathological and immunohistochemical results. Future histopathological studies may be necessary to develop more specific markers aimed at optimizing the differential diagnostic process. On the other hand, imaging examinations are also highly beneficial for the diagnosis of ANNUBP. Current studies support the utility of PET-CT and MRI in evaluating surgical complications associated with the marginal resection of atypical neurofibromas (18). Tovmassia et al. (19) concluded that there is a significant difference in the average maximum standardized uptake value (SUVmax) between benign and malignant tumors in NF1 patients; however, a definitive SUVmax cutoff value remains to be established. Investigating the imaging characteristics of ANNUBP for early diagnosis represents a promising avenue for future research.

Tumors that develop in the nasal cavity can cause symptoms such as nasal congestion, bloody secretions, and loss of smell in the early stages (20). These tumors are typically smaller at the time of diagnosis compared to tumors in other parts of the body, making early detection and treatment easier (21). Endoscopic surgery has become the primary method for treating benign nasal tumors due to its proven effectiveness (20, 22). Both patients in this study underwent standard endoscopic tumor resection, and no recurrence was observed during the postoperative follow-up. While the optimal extent of surgical resection for ANNUBP has yet to be established, it is widely accepted that complete surgical resection is the primary treatment approach for benign nasal tumors (20). Currently, there is no reliable clinical study that defines the appropriate scope of surgical resection for ANNUBP. In contrast, for pNF, surgical resection remains the preferred treatment option (23). For MPNST, extended tumor resection—which involves the removal of the tumor along with 3 cm of surrounding barrier tissue—is currently regarded as the optimal treatment option (18). Currently, there is no consensus on the treatment options for ANNUBP. Most approaches advocate for complete surgical resection. Given its unique disease progression, further discussion is warranted regarding whether the extent of resection should be expanded in reference to the treatment methods employed for MPNST. Bernthal et al. (24) demonstrated that patients with ANNUBP who underwent marginal resection did not experience metastasis or disease-related death, even when the resection margin was involved. Current research indicates that ANNUBP has a low risk of recurrence and an almost negligible risk of metastasis (3); therefore, we consider its prognosis to be superior to that of MPNST. Meantime, there are no studies indicating that ANNUBP exhibits invasive growth characteristics (1), suggesting that extensive clearing of mucosal and bone structures may not be necessary.

While radiotherapy is effective in achieving local control and delaying the recurrence of MPNST, it does not significantly enhance the long-term survival rate (25). Chemotherapy recommendations adhere to those established for other soft tissue sarcomas. These include, but are not limited to, liposomal doxorubicin monotherapy (Doxil), doxorubicin and ifosfamide with mesna (AIM), and irinotecan combined with carboplatin and etoposide (ICE), doxorubicin with ifosfamide and dacarbazine, or doxorubicin and cyclophosphamide (11, 26); Although some studies have indicated that the therapeutic effects of chemotherapy on MPNST are quite limited, both clinically and histologically (9). To date, no targeted treatment options for MPNST have been demonstrated to be effective. However, some evidence suggests a potential role for immunotherapy in the treatment of MPNST (11). Currently, several clinical drug trials for NF1-related pNF have been conducted, and some drugs have received approval for use in patients (11, 27). The findings from these studies will also serve as valuable references for the investigation of additional treatment options for ANNUBP. We anticipate new breakthroughs in research across various fields, including chemotherapy and immunotherapy, aimed at further optimizing treatment strategies for ANNUBP and other neurofibromas.

## Conclusion

We presented two cases of patients with ANNUBP occurring in the nasal cavity. Standard endoscopic tumor resection surgeries were performed for both patients in accordance with the principle of complete resection, resulting in significant curative effects. We emphasize the necessity of long-term follow-up to monitor for recurrence or malignant transformation of the tumors.

## Data Availability

The raw data supporting the conclusions of this article will be made available by the authors, without undue reservation.
